# The TNFα-Transgenic Rat: Hippocampal Synaptic Integrity, Cognition, Function, and Post-Ischemic Cell Loss

**DOI:** 10.1371/journal.pone.0154721

**Published:** 2016-05-04

**Authors:** L. Creed Pettigrew, Richard J. Kryscio, Christopher M. Norris

**Affiliations:** 1 Paul G. Blazer, Jr. Stroke Research Laboratory, University of Kentucky, Lexington, Kentucky, United States of America; 2 Sanders-Brown Center on Aging, University of Kentucky, Lexington, Kentucky, United States of America; 3 Department of Neurology, University of Kentucky, Lexington, Kentucky, United States of America; 4 Veterans Administration (VA) Medical Center, Lexington, Kentucky, United States of America; 5 Department of Statistics and School of Public Health, University of Kentucky, Lexington, Kentucky, United States of America; 6 Department of Pharmacology and Nutritional Sciences, University of Kentucky, Lexington, Kentucky, United States of America; Emory University, UNITED STATES

## Abstract

The cytokine, tumor necrosis factor α (TNFα), is a key regulator of neuroinflammation linked to numerous neurodegenerative conditions and diseases. The present study used transgenic rats that overexpress a murine TNFα gene, under the control of its own promoter, to investigate the impact of chronically elevated TNFα on hippocampal synaptic function. Neuronal viability and cognitive recovery in TNFα Tg rats were also determined following an ischemic insult arising from reversible middle cerebral artery occlusion (MCAO). Basal CA3-CA1 synaptic strength, recorded in acute brain slices, was not significantly different between eight-week-old TNFα Tg rats and non-Tg rats. In contrast, slices from TNFα Tg rats showed significantly greater levels of long-term potentiation (LTP) in response to 100 Hz stimulation, suggesting that synaptic networks may be hyperexcitable in the context of elevated TNFα. Cognitive and motor deficits (assessed on the Morris Water Maze and Rotarod task, respectively) were present in TNFα Tg rats in the absence of significant differences in the loss of cortical and hippocampal neurons. TNF overexpression exacerbated MCAO-dependent deficits on the rotarod, but ameliorated cortical neuron loss in response to MCAO.

## Introduction

Tumor necrosis factor-α (TNFα) is a pleiotropic cytokine suspected to enhance or deter cellular survival through activation of receptor-mediated signal transduction. When present at supra-physiological levels after injury, it is known to modulate neural cell loss in cerebral ischemia [1], intracerebral hemorrhage [2], chronic cerebral oligemia [3], and trauma [4]. The level of TNFα in human brain becomes elevated after cerebral infarction [5] and appears sequentially in the infarct core and peri-infarct areas before expression in tissue within the unaffected hemisphere [6]. Elevated levels of TNFα have been observed consistently in serum [7–9] and in cerebrospinal fluid [8, 9] after acute ischemic stroke. In animal models of cerebral ischemia, high levels of TNFα have been found after global [10, 11] and focal [12] ischemic injury. Several investigators reported reduction of infarct volume through inhibition of TNFα [13–15], although Nawashiro and colleagues [16] showed that pretreatment of mice by intra-cisternal administration of TNFα reduced infarct volume paradoxically without an inhibitor. Anti-TNFα antibodies have been shown to be neuroprotective and may reduce infarct volume in focal ischemic models by as much as 85% [13, 17, 18].

Although TNFα is well recognized as an inflammatory mediator that may enhance neuronal loss after cerebral ischemia, recent evidence suggests that it may have an alternate, beneficial role in augmenting neural recovery. This multi-faceted capacity of the cytokine appears to be driven by complex interactions between TNFα in its active, soluble form, its less active precursor, and its principal receptors on mammalian cells, p55/tumor necrosis factor-receptor 1 (TNF-R1) and p75/TNF-R2 [19, 20]. Some of the most intriguing reports have been in regard to TNF receptor-mediated regulation of neurogenesis. Iosif and colleagues [21] demonstrated that TNF-R1^-/-^ or TNF-R1/R2^-/-^ mice had enhanced expression of mature hippocampal neurons *in vivo*, an effect that was not observed in TNF-R2^-/-^ mice. The same investigators further pursued the seemingly unique role of TNF-R1 in the regulation of progenitor cell growth after cerebral ischemia [22]. They demonstrated that progenitor cell proliferation would be enhanced within the subventricular zone (SVZ) in the brain of TNF-R1^-/-^ mice subjected to middle cerebral artery occlusion (MCAO). Furthermore, addition of TNFα to cultured neurospheres harvested from the SVZ decreased cellular growth through a TNF-R1-mediated mechanism, without causing cell death. These findings suggest that TNF-R1 is a negative regulator of SVZ progenitor growth and indicate that a selective antagonist of this receptor, if administered effectively *in vivo*, may augment neural cell replacement after ischemia.

To complement its dualistic roles in cell recovery or death after physiological stress, TNFα is recognized as an important contributor to neurotransmission and synaptic plasticity. Glial TNFα can cause an increase in cell-surface expression of excitatory, neuronal α-amino-3-hydroxy-5-methyl-4-isoxazole propionic acid (AMPA) receptors [23, 24], which would improve synaptic efficacy and/or increase neuronal excitability. This TNFα-induced AMPA receptor exocytosis has been shown to be mediated by activation of TNF-R1 through a phospho-inositol triphosphate (PI3) kinase-dependent pathway [25]. The same action of glial TNFα can also lead to endocytosis of inhibitory gamma-aminobutyric acid (GABA) receptors. TNFα is also known to act as a regulator of synaptic plasticity in the dentate gyrus of the hippocampus. TNFα knock-out mice show reduced dendritic arborization in the CA1 and CA3 regions while at the same time have accelerated development within the dentate gyrus [26], probably by ligand-mediated activation of TNF-R2. Patho-physiological levels of TNFα in the CA1 region and in the dentate gyrus are known to inhibit long-term potentiation (LTP; [27–29], a long-lasting increase in synaptic efficacy that is considered to be an important underlying mechanism of learning and memory formation [30]. Butler and colleagues [31] found that impairment of early-phase (<120 minutes post-tetanic stimulation) LTP by TNFα was dependent upon activation of mitogen-activated protein kinase (MAPK) p38. Inhibition of p38 MAPK with a selective antagonist blocked the impairment of early-phase LTP by TNFα, but had no effect on late-phase (>120 minutes post-tetanic stimulation) LTP. However, TNFα-mediated inhibition of LTP is not dependent on the presence of active TNF-R1/2, as observed by Albensi and Mattson [32] who found that LTP could be stimulated in hippocampal slices taken from TNF-R1/R2^-/-^ mice.

In the present experiments, we sought to determine the effect of constitutive upregulation of TNFα synthesis on hippocampal synaptic plasticity and on cognitive and functional performance after ischemic brain injury. Here, we used TNFα-transgenic (TNFα-Tg) rats that over-express the murine TNFα gene from its native promoter. In these animals, levels of biologically active TNFα in brain tissue are 5-fold higher than in non-Tg littermates [33]. In our previous studies [33], we demonstrated that TNFα-Tg rats have increased caspase 3 activity within the infarct core and ischemic penumbra and are more susceptible to apoptotic cellular death after 24 hours of reperfusion, when compared to non-Tg littermates. The overall goal of the current study was to determine if increased levels of TNFα protein in cerebral tissue, as may be found in human brain after ischemic stroke, will exacerbate memory impairment after ischemia and contribute to the risk of vascular cognitive impairment (VCI). Utilizing the novel resource of the TNFα-Tg rat, we tested the hypothesis that elevated brain levels of TNFα protein will undermine hippocampal synaptic integrity or contribute to enhanced neuronal loss after cerebral ischemia, thereby worsening cognitive or functional impairment.

## Methods

### Ethics Statement

All experimental methods using animal subjects were approved by the Institutional Animal Care & Use Committee of the University of Kentucky in Lexington, Kentucky. Every effort was made to provide ethical, humane and compassionate care for animals during experimental procedures, in full observance of federal guidelines and of recommendations issued by the American Veterinary Medical Association.

### Construction and Genotyping Transgenic Rats

Construction of the TNF-Tg rat and its detailed characterization have been described [33]. All rat pups underwent genotyping from tail-snip tissue at 21 post-natal days, to confirm the presence or absence of the murine TNFα transgene construct [33]. Hemizygous TNFα-Tg rats expressed the murine TNFα gene and its native promoter; affected animals had elevated levels of TNF mRNA and biologically active TNF protein in brain tissue [33].

### Electrophysiologic Analysis of Hippocampal Synaptic Integrity

Methods for preparing hippocampal slices and recording synaptic responses in area CA1 of rat hippocampus have been reported elsewhere in greater detail [34–37]. All recordings of synaptic function were performed by an examiner blinded to genotype conditions (C.M.N.).

TNFα-Tg rats and non-Tg littermates (and in some cases, two-to-three week-old non-Tg rats) were euthanized in a CO_2_ chamber and decapitated. Brains were removed *en bloc* and stored briefly in ice-cold, oxygenated (95% O_2_, 5% CO_2_) artificial cerebrospinal fluid (ACSF) that contained (in mM) 124 NaCl, 2 KCl, 1.25 KH_2_PO_4_, 2 MgSO_4_, 0.5 CaCl_2_, 26 NaHCO_3_, and 10 dextrose at pH ~ 7.4. Hippocampi were removed and sliced (450 μm sections) parallel to the alvear fibers using a McIlwain tissue chopper. Slices were then transferred to netting in a custom plexiglass holding chamber [38] and bathed in recording medium (oxygenated ACSF containing 2 mM CaCl) at an interface with humidified air. Slices equilibrated for at least 1.5 h before transfer to a modified RC-22 recording chamber (Warner Instruments, Hamden, CT) secured to the stage of a Nikon E600FN microscope where they were perfused with recording medium (32°C) at a rate of 1–2 mL/min.

The recording electrode, consisting of a glass pipette (~8 MΩ resistance) filled with ACSF and a silver chloride wire, was positioned extracellularly in *stratum radiatum* of area CA1. Field EPSPs were elicited by diphasic (100 μs) current pulses delivered through a bipolar platinum/iridium wire positioned in *stratum radiatum* near the CA3 border. Stimulus intensity was controlled by a constant current stimulus isolation unit (World Precision Instruments). At the outset of each recording session, a full input/output (I/O) curve was constructed using nine stimulus intensity levels (30, 50, 100, 150, 200, 250, 300, 400, and 500 μA), with five field EPSPs elicited at each level at a rate of 0.1 Hz. Twin diphasic pulses at each stimulus level, separated by a 50 ms interpulse interval, were used to assess paired-pulse facilitation (PPF). After the I/O curve, stimulus intensity was adjusted to elicit an approximately 1 mV field EPSP and single stimulus pulses were delivered at a rate of 0.033 Hz. LTP was induced using two 100 Hz stimulus trains (1 s duration) separated by 10 s at baseline stimulus intensity. EPSPs were then followed for at least 60 minutes after LTP-induction.

### Field potential parameters and measures of synaptic strength and plasticity

For I/O curves, the presynaptic fiber volley amplitude (mV) was plotted against the stimulation intensity level to determine fiber excitability levels. EPSP slope, calculated as the difference between two cursors spaced 1 ms apart on the middle of the descending phase of the EPSP, was measured to determine CA3-CA1 monosynaptic response magnitudes. EPSP slopes were plotted against the respective fiber volley amplitudes to determine relative synaptic strength. Paired pulse facilitation, determined from the delivery of twin stimulus pulses (see above), was indicated as the percent change in the EPSP slope following pulse two relative to the EPSP slope following pulse one. For LTP experiments, EPSP slopes were averaged during the last 10 minutes of the post-100 Hz baseline and expressed as a percentage of the average EPSP slope during the last 10 minutes of the pre-100 Hz baseline.

### Electrophysiologic Data Acquisition

Field potentials were amplified 100X, bandpass-filtered between 1 Hz and 1 kHz by a Multiclamp 700B amplifier (Axon Instruments), converted to digital units, and stored on a computer for off-line analysis. Stimulus timing and data acquisition were controlled by pClamp Ver.9 software (Axon Instruments).

### Focal Cerebral Ischemia

Because the murine TNFα gene was expressed within Sprague-Dawley rats, the Zea Longa technique [39] was selected for suture-occlusion of the MCA. Male transgenic and non-Tg rats of 275–325 gm body weight were subjected to one hour of suture-occlusion by our modification [40] of the original Zea Longa method. Each animal was fasted overnight in preparation for surgery and then anesthetized by IP injection of chloral hydrate (350 mg/kg) and xylazine (4 mg/kg). Rectal and temporalis muscle temperatures were maintained at 36.5–37.5°C by external warming. A suture-occluder prepared by the method of Belayev and others [41] was advanced retrograde through the external carotid artery and into the internal carotid artery to occlude the MCA. After one hour, the suture-occluder was withdrawn.

### Assessment of Reference Memory by Water Maze

Both TNFα-transgenic and non-Tg rats underwent assessment of reference memory performance using the Morris water maze technique [42–44]. All animals were trained in the water maze before MCAO and were tested after seven days of post-ischemic recovery. Rats were trained and assessed in a circular tank (170 cm diameter, 56 cm height) that was painted black, filled with water maintained at 27°C, and placed in a well-lit room with black walls. A circular goal platform of clear Plexiglass (13 cm diameter) was placed with the surface lying approximately 1.5 cm below water level in the tank. Non-toxic black powder paint was added to the water to further obscure the goal platform. For each series of training blocks, the goal platform was located randomly within one of 4 quadrants in the tank and remained constant relative to visual cues placed in the testing room. A video camera mounted directly above the center of the tank recorded swimming performance. Each record was analyzed by a video motion analyzer (Videomax I, Columbus Instruments, Columbus, OH).

Animals were habituated to the tank by 30 s of free swim. Training consisted of 5 blocks of 3 trials per block, with all training completed within one day. Inter-trial intervals were 20 s and inter-block intervals were 15 minutes. Each trial began by manually releasing the rat into the water from one of four start locations (N, S, E, and W) spaced at equivalent distances along the perimeter of the tank. During each trial, the rat was allowed 60 s to find and ascend the goal platform. If the animal did not escape within 60 s, then it was directed to the platform. Acquisition goal latency was defined as the time elapsed between the release of the animal into the tank and its successful ascension of the hidden platform. Rats that were unable to ascend the goal platform were given an goal latency of 60 sby definition. Each animal remained on the platform between trials and was warmed in a heated incubator between blocks. Fifteen minutes following the end of training, a free-swim probe trial was administered in order to test reference memory. Probe trials consisted of placing the animal in the tank for one minute without the platform and recording the time the animal spent in each quadrant [42].

### Assessment of Functional Performance by Rotarod

Transgenic rats and non-Tg littermates were tested serially for motor function before and for 28 days after focal cerebral ischemia using a Rotarod (Economex; Columbus Instruments, Columbus, OH), with slight modfications described by Candelario-Jalil and colleagues [45]. The rotating rod was fitted with dividers to accommodate rats. For each animal, training began on the day of surgical preparation for MCAO. On the first training day, the rat was acclimated to the Rotarod device by being placed on the stationary rod for 120 s. Animals unable to remain in place on the stationary rod for this duration were repositioned serially until able to do so. After the rat was acclimated, it was removed from the rod for a one-minute rest period. The rat was then repositioned on the stationary rod for an acceleration trial that began at 2 revolutions per minute (RPM) and increased linearly to 20 RPM within 300 s. Each animal was required to remain on the moving rod for a minimum fall latency (FL) of 30 s. If the animal was unable to meet this criterion, then the acceleration trial was repeated up to five times with one-minute rest periods in between. If up to five trials were required for the animal to remain on the moving rod for the 30 s minimum, then the two longest FLs were averaged as the pre-ischemic baseline. If the rat remained on the rotating rod for the entire 300 s duration, then this time was assigned as the pre-ischemic FL.

RotaRod sessions were undertaken at 24 and 48 hours after reversal of MCAO (or after sham surgery), and twice weekly thereafter on weeks 2–4. The twice-weekly examinations were separated by two days. The last examination did not exceed 28 days from the date of ischemia by MCAO. Beginning with the 24-hour post-ischemic examination, each rat was placed on the stationary rod and had to maintain this position for a minimum of 30 s before being advanced to an acceleration trial. If the animal remained in place on the stationary rod for 120 s, then this value was defined as the maximum score in the stationary position. Once the animal remained in place on the stationary rod for a minimum of 30 s, it would be rested for one minute before beginning an acceleration trial (2–20 RPM within 300 s) to define FL. For each date of post-ischemic examination, the best two FLs determined from a minimum of three trials were averaged for an overall performance score. The animal was allowed one minute of rest between each attempt.

### Stereological Analysis in Hippocampus and Cortex

After completion of the 28-day Rotarod experiment, animals were euthanatized for removal of brain *en bloc*. Quantitative stereological analysis of neuronal preservation in the hippocampus and ischemic cortex was performed as described previously [46–49]. Each whole brain was immersed in OCT Embedding Compound for cryostat sectioning and 30-μm frozen coronal sections were collected. Each section contained the ischemic and contralateral, unaffected hemispheres. The hippocampus was sampled in an anterior to posterior direction (3.3 to 4.3 mm posterior to bregma). Every 20^th^ section was mounted on pre-cleaned Fisher SuperfrostTM microscope slides. The resulting sections were Nissl-stained with 1% cresyl violet, according to a standard histological protocol.

Neuronal counting was performed in the selectively vulnerable CA1 and CA3 regions of the dorsal hippocampus and in the overlying cortex. Pyramidal neurons in CA1 and CA3 of the hippocampus and in the cortex were imaged sequentially using a 40 × objective (Olympus OX-40 microscope). The CA1 region was identified as the layer consisting of small pyramidal neurons in the dorsal hippocampus. The CA3 region was identified as the layer of large pyramidal neurons in the ventral hippocampus extending from the dentate gyrus to the transition zone adjacent to CA1. Pyramidal neurons in layer 5 of the cortex were identified and cell counting was performed in both hemispheres. Manual counting of the neurons in each subfield was performed using Infinity Camera1 and the analysis was done using 5.0.6 software (Lumenera Corporation). All cells, regardless of the intensity of staining, were counted within defined sample grids (100 × 100 μm^2^ for the hippocampus and 400 × 400 μm^2^ for the cortex); four grids were counted in each region. Only histologically normal-appearing neurons with clearly defined cell bodies and nuclei were counted. Neurons that were partially obscured due to the level of sectioning were not included. Three adjacent coronal sections were analyzed from each animal. Mean values were calculated for these regions of interest, as previously described in the procedure. Each cresyl violet-stained section was analyzed by two independent investigators who were blinded to the experimental design.

### Statistical Analysis

All electrophysiological data acquired during recordings from hippocampal slices were expressed as means ± SEM and were analyzed using Statview Ver. 5. For measurements of synaptic function, data were obtained in at least two brain slices per animal. Long-term potentiation and PPF values across slices were then averaged to yield a single data point from each animal for every measurement, such that sample sizes equaled the number of animals per group defined by genotype.

For analysis of reference memory testing in the water maze, Fisher’s exact test was employed to detect differences between AGLs in the first and last blocks of trials and repeated measures ANOVA was performed to test for differences in AGL reduction over time. To evaluate probe trial data, one-sample *t*-tests were used to compare the percent search time in specific quadrants of the water maze relative to chance (i.e., 25%).

For statistical analysis of Rotarod data, group mean responses were compared by constructing a linear mixed model for a 2 x 2 factorial design (TNFα-Tg or non-Tg groups; MCAO or sham-ischemia). Repeated measurements were obtained from each animal, beginning 24 hours after MCAO or sham-ischemia and concluding on post-ischemia Day 28. The dependent variable was FL. Since the variability in this measurement was considerable across time, a univariate structure was adopted for the correlation matrix of the repeated measurements. Degrees of freedom for the F tests and all comparisons among group means were estimated by Satterthwaite’s procedure.

For statistical analysis of hippocampal and cortical histology at 28 days after focal ischemia, cell counts were pooled by region (CA1, CA3, and cortex layer 5) and by experimental condition (hemispheric laterality and sham v. ischemic) for one-way ANOVA. When appropriate after confirmation of a statistically significant primary effect by ANOVA, *post hoc* analysis was performed with Tukey’s test for multiple comparisons. (*p* < 0.05).

## Results

### TNFα-rats show alterations in hippocampal synaptic plasticity

**Fig 1:**
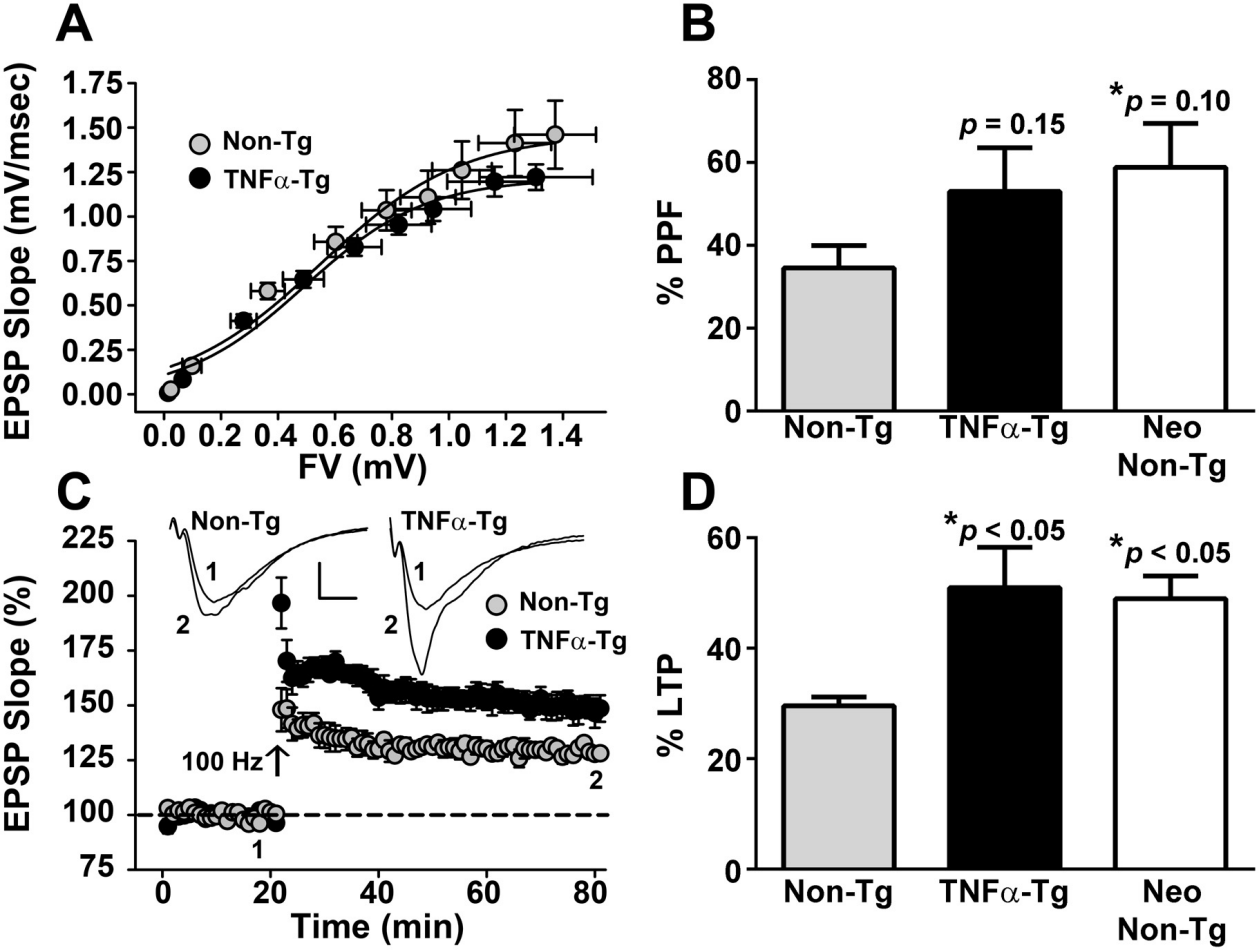
Measurement of CA3-CA1 synaptic function in hippocampal slices from TNFα-Tg and non-Tg rats. ***A***, Mean ± SEM CA1 excitatory postsynaptic potentials (EPSPs) in slices from adult Non-Tg and TNFα-Tg rats (*n* = 6-8/group) plotted against the CA3 fiber volley (FV) amplitude generated from nine successively increasing stimulus intensities. The maximal EPSP slope was slightly, though insignificantly, increased in non-Tg hippocampus. ***B***, Mean ± SEM paired-pulse facilitation (PPF) of the EPSP is shown in response to twin stimulus pulses administered with a 50 ms inter-pulse interval. Non-Tg neonatal (Neo) rats (2–3 weeks-old, *n* = 5) are shown for comparison. PPF was greater (albeit, nonsignificantly) in TNFα-Tg rats and Non-Tg neonatal rats relative to Non-Tg adult rats (*p* > 0.05). **C**, Time plot showing normalized mean ± SEM EPSP slope amplitudes in slices from adult Non-Tg and TNFα-Tg rats (*n* = 6-8/group) before and after the delivery of two 100-Hz trains (arrow) to induce LTP. *Inset* shows representative EPSP waveforms averaged during the last 10 minutes of the pre-LTP (1) and post-LTP (2) baselines (calibration bars, 0.5 mV/5 ms). D, Mean ± SEM LTP measured 60 min after the delivery of 100 Hz stimulation. Non-Tg neonatal (Neo) rats (2–3 weeks-old, *n* = 5) are shown for comparison. LTP was significantly elevated in TNFα-Tg rats and Non-Tg neonatal rats relative to Non-Tg adult rats.

We first determined if chronic overexpression of TNF alters synaptic function and plasticity in the hippocampus, a structure that is essential for cognitive function and is highly vulnerable to secondary injury from MCAO. Field potentials were examined in CA1 *stratum radiatum* of hippocampal slices obtained from naïve TNFα-Tg rats and non-Tg littermates (*n* = 6–8 per group) that did not undergo focal cerebral ischemia. All animals were examined at 8 weeks of post-natal age, to maintain consistency with other studies performed in rats subjected to MCAO (see below). Measures of fiber volley (FV) amplitude and excitatory post-synaptic potential (EPSP) slope were taken in response to electrical stimulation of Schaffer collaterals using our standard methods [36–38, 50, 51]. As depicted in Fig 1A, synaptic strength curves appeared very similar across genotypes, indicating that both fiber excitability and basal synaptic strength are not appreciably affected by chronically elevated TNFα. In contrast, paired-pulse facilitation (PPF), a transient form of synaptic plasticity, and long-term potentiation (LTP), a long-lasting form of plasticity, were both increased by a similar extent in TNFα Tg rats (Fig 1B–1D). LTP differences reached statistical significance (51 ± 7.3% *vs* 29.6 ± 1.6%, *p* < 0.05), while PPF did not (53 ± 10.5% *vs* 34.6 ± 5.4%, *p* = 0.15), due to overall greater variability. Elevated PPF and LTP levels at hippocampal synapses have also been reported in neonatal (< 3 weeks old) relative to adult rats [52, 53]. When we investigated CA3-CA1 synaptic function in two-three week old Non-Tg rats (*n* = 5), we observed PPF and LTP levels that were comparable to adult TNFα Tg rats (PPF 58.9 ± 10.6%, *p* > 0.05; LTP 49 ± 4.1%, *p* > 0.05), but markedly greater than that shown by adult Non-Tg rats (PPF *p* = 0.1; LTP *p* < 0.05). Together, these observations suggest that chronically elevated TNFα may retard the maturation of hippocampal circuitry.

### TNFα-rats show mild spatial cognition deficits

**Fig 2:**
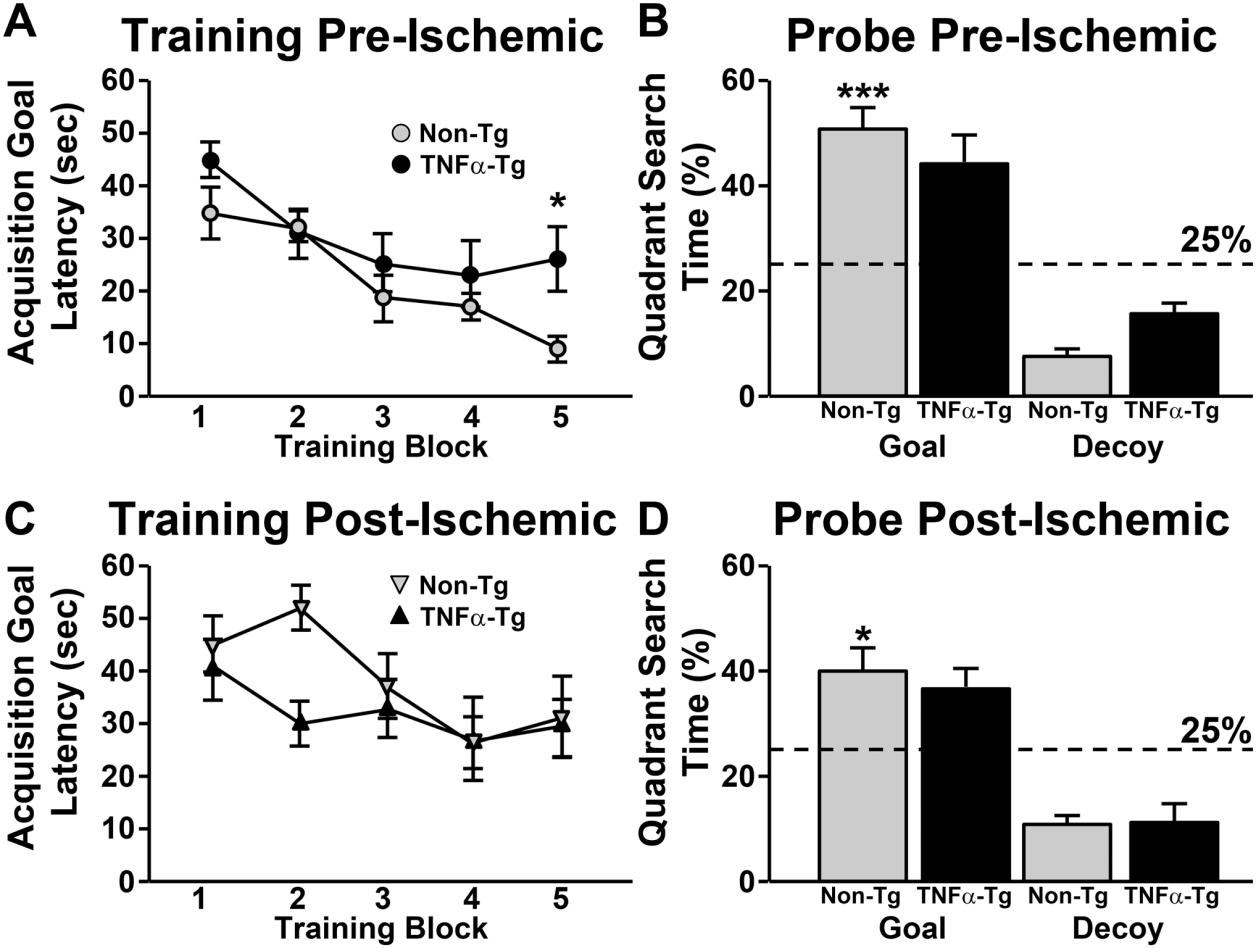
Spatial learning in the Morris Water Maze before and after MCAO. Five training blocks (3 trials/block) were given across one day on the Morris Water Maze (***A***, ***C***), followed by a free-swim probe trial (***B***, ***D***). ***A***, Mean acquisition goal latency (AGL) ± SEM in seconds (averaged across 3 trials within each block) for TNFα-Tg and non-Tg littermates prior to MCAO (*n* = 10–13 rats per genotype/treatment group). Significant reductions in AGL were observed over training in both groups, with non-Tg mice outperforming TNFα-Tg mice by block 5. ***B***, Mean percent time ± SEM spent searching the goal quadrant or a decoy quadrant (opposite the goal) during the free-swim probe prior to MCAO. The time spent by non-Tg mice in the goal quadrant was significantly above chance levels (i.e. 25%), (***p < 0001). In contrast, the percent time spent by TNFα-Tg mice in the goal quadrant did not differ significantly from chance. *C*, AGL ± SEM on the Morris Water maze assessed seven days after MCAO. Neither TNFα-Tg nor non-Tg rats showed significant reductions in AGL across training. *D*, Mean percent time ± SEM spent searching the goal and decoy quadrants at seven days post-MCAO. Non Tg rats continued to search above chance levels in the goal quadrant (*p < 0.05). Conversely, goal search times for TNFα-Tg rats did not differ from chance levels.

Reference memory performance of TNFα-Tg and non-Tg rats in a spatial discrimination task is shown in Fig 2 (*n* = 10–13 per group). All animals were assessed with the Morris water maze before (Fig 2A and 2B) and seven days after (Fig 2C and 2D) MCAO. Testing consisted of five training blocks of three trials followed by a “free-swim” probe trial. The time to mount an escape platform, or the acquisition goal latency (CGL) during each training block and the percent time spent searching the goal quadrant during the 60 s probe trial were used as indices for spatial learning.

Prior to MCAO, both TNFα-Tg and non-Tg rats exhibited learning across training (Fig 2A), as shown by statistically significant reductions in AGLs over the first to the fifth block of trials (TNFα-Tg rats, *p* < 0.01; non-Tg rat, *p* < 0.001). However, by the fifth training block, TNFα-Tg rats showed significantly slower escape times relative to the non-Tg group (*p* ≤ 0.05). On the probe trial, the time spent searching the goal quadrant for non-Tg rats was significantly greater (*p* < 0.0001, one sample *t*-test) than what would be expected if a random search strategy was used (i.e. 25% time in the goal quadrant). In contrast, the time spent in the goal quadrant for TNFα-Tg rats was more variable and did not differ significantly from chance. TNFα-Tg rats also spent more time than non-Tg rats searching the “decoy” quadrant, (i.e. the quadrant opposite to the goal quadrant), consistent with poorer spatial learning.

When the same rats were tested on the Morris Water Maze 7 days after MCAO, neither group showed significant improvement (*i*.*e*. faster AGLs) over training (Fig 2C), suggesting that MCAO caused learning impairments for both genotype groups. Time spent searching the goal quadrant in the probe trial (Fig 2D) also went down for both groups following MCAO (*i*.*e*. compare search times in Fig 2D and 2B). Nonetheless, non-Tg rats continued to target the goal quadrant above chance levels (vs. 25%, *p* < 0.05), while TNFα-Tg rats did not. Together these results indicate that the overexpression of TNF is associated with a mild hippocampal-dependent cognitive deficit. However, it does not appear that ischemia-dependent cognitive changes are worsened by TNF.

### TNFα exacerbates motor deficits following MCAO

**Fig 3:**
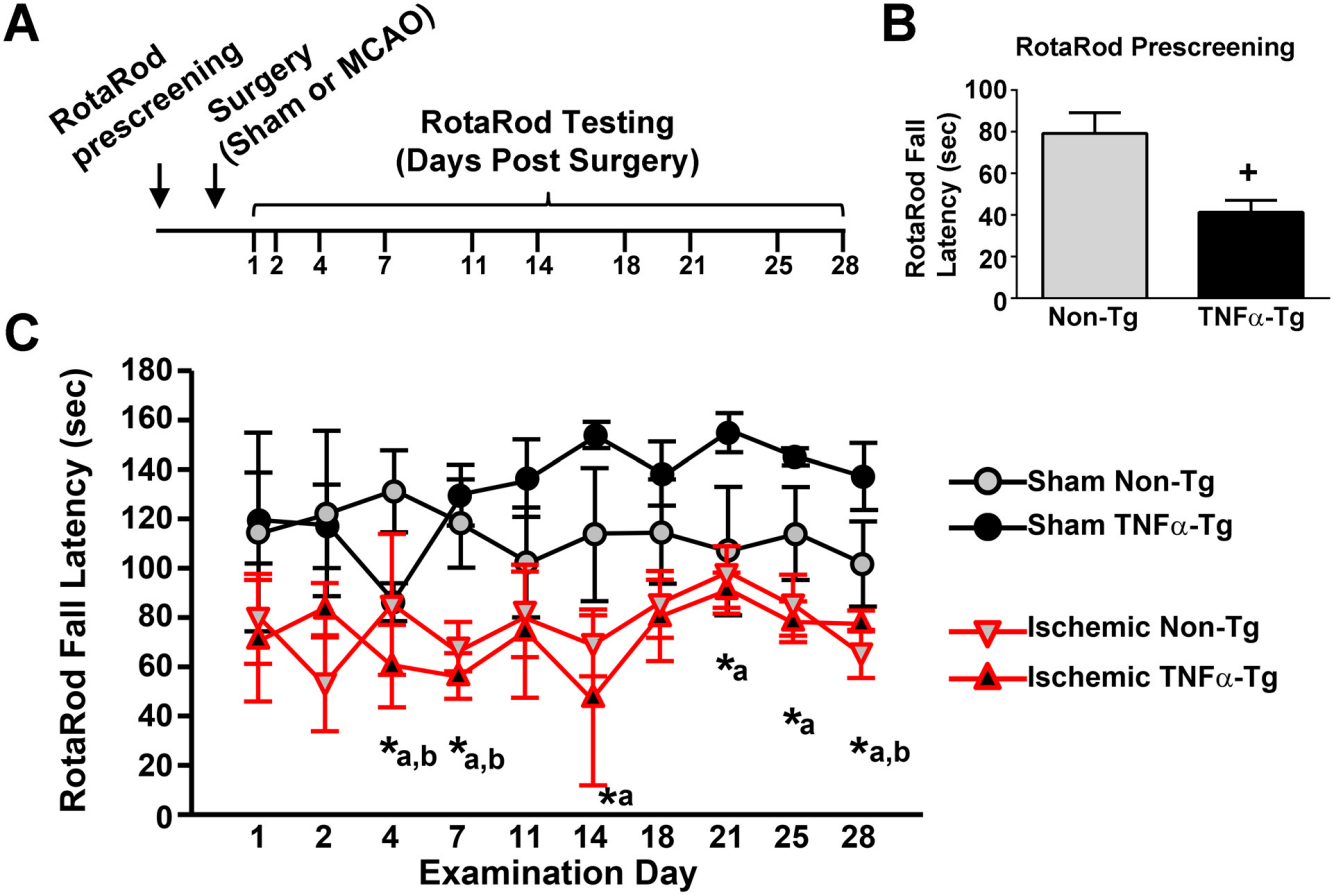
Fall latency time in a Rotarod task performed by TNFα-transgenic and non-transgenic rats. *A*, Experimental time line for surgical procedures and Rotarod testing. Non-Tg (*n* = 9) and TNFα-Tg (*n* = 7) rats were pre-screened on a Rotarod task and then randomly assigned to receive sham surgery or MCAO to induce cerebral ischemia. Rats were then tested again on the Rotarod at 1, 2, 4, 7, 11, 14, 18, 21, 25, and 18 days post-surgery. *B*, Mean ± SEM fall latency on the Rotarod prescreening test (*i*.*e*. prior to surgery). ^+^*p* < 0.01. *C*, Multi-line plot shows mean ± SEM fall latency (FL) during post-surgery Rotarod testing (MCAO, high-lighted in red) or sham-surgery. Significant differences between mean fall latencies for ischemic and sham-operated TNFα-Tg rats (*n* = 4 and 3, respectively) were found on Days 4, 7, 14, 21, 25, and 28. For non-Tg littermates, significant differences between ischemic and sham-operated rats (*n* = 5 and 4, respectively) were observed only on Days 4, 7, and 28. *p ≤ 0.05; a = *post hoc* comparison between mean FLs of ischemic and sham-operated TNFα-Tg rats; b = *post hoc* comparison between mean FLs of ischemic and sham-operated non-Tg rats.

To determine the effects of chronically elevated TNFα on motor function, non-Tg and TNFα-Tg rats were tested on the Rotarod then randomly assigned to receive MCAO or sham surgery (Fig 3A). Prior to surgery, each animal was able to maintain its balance on the stationary rod for 120 s. Fall latency (FL) was defined by averaging the longest two durations achieved by each animal after five trials on the accelerating rod. Prior to MCAO or sham surgery, the mean ± SEM FL for TNFα-Tg rats (41.3 ± 5.7 s; *n* = 7) was significantly shorter than that of non-Tg animals (79.2 ± 9.9 s; n = *9*; unpaired *t*-test; *p* ≤ 0.01, Fig 3B). Data were then collected from serial, accelerating trials on the Rotarod on Days 1, 2, 4, 7, 11, 14, 18, 21, 25, and 28 after surgery (Fig 3C), with data from ischemic animals (*i*.*e*. animals receiving MCAO) highlighted in red. Using a linear mixed model, we detected a significant interaction between injury (MCAO vs sham), genotype (TNFα-Tg vs non-Tg), and date of examination after surgery (*p* ≤ 0.05). Although the pre-surgery FL differed significantly between the TNFα-Tg and non-Tg groups (Fig 3B), sham animals in both genotype groups performed similarly over the 28-day post-surgery interval. Ischemic TNFα-Tg rats (*n* = 4) had FLs of shorter duration compared to TNFα-Tg sham-operated rats (*n* = 3) on Days 4, 7, 14, 21, 25, and 28 (*p* ≤ 0.05 on each Day, see Fig 3C and Table 1). In contrast, non-Tg ischemic rats (*n* = 5) differed significantly from sham-operated non-Tg sham rats (*n* = 4) on only three examinations (Days 4, 7, and 28; *p* ≤ 0.05 each, see Fig 3C and Table 1). There was no significant difference in FL between TNFα-Tg and non-Tg ischemic rats over 28 days. Overall, TNFα-Tg rats exposed to ischemia had shorter mean FLs, indicating greater impairments in the Rotarod task when compared to TNFα-Tg sham-operated rats, for twice as many days as were observed with non-Tg rats.

**Table 1:** *Post Hoc* Analyses of RotaRod Fall Latencies (Sham *vs* MCAO-ischemic).

Days	1	2	4	7	11	14	18	21	25	28
**Non-Tg**	*n*.*s*	*n*.*s*	*p*<0.05	*p*<0.05	*n*.*s*	*n*.*s*	*n*.*s*	*n*.*s*	*n*.*s*	*p*<0.05
**TNFα-Tg**	*n*.*s*	*n*.*s*	*p*<0.05	*p*<0.05	*n*.*s*	*p*<0.05	*n*.*s*	*p*<0.05	*p*<0.05	*p*<0.05

### TNFα alleviates cortical neuron loss following MCAO

**Fig 4:**
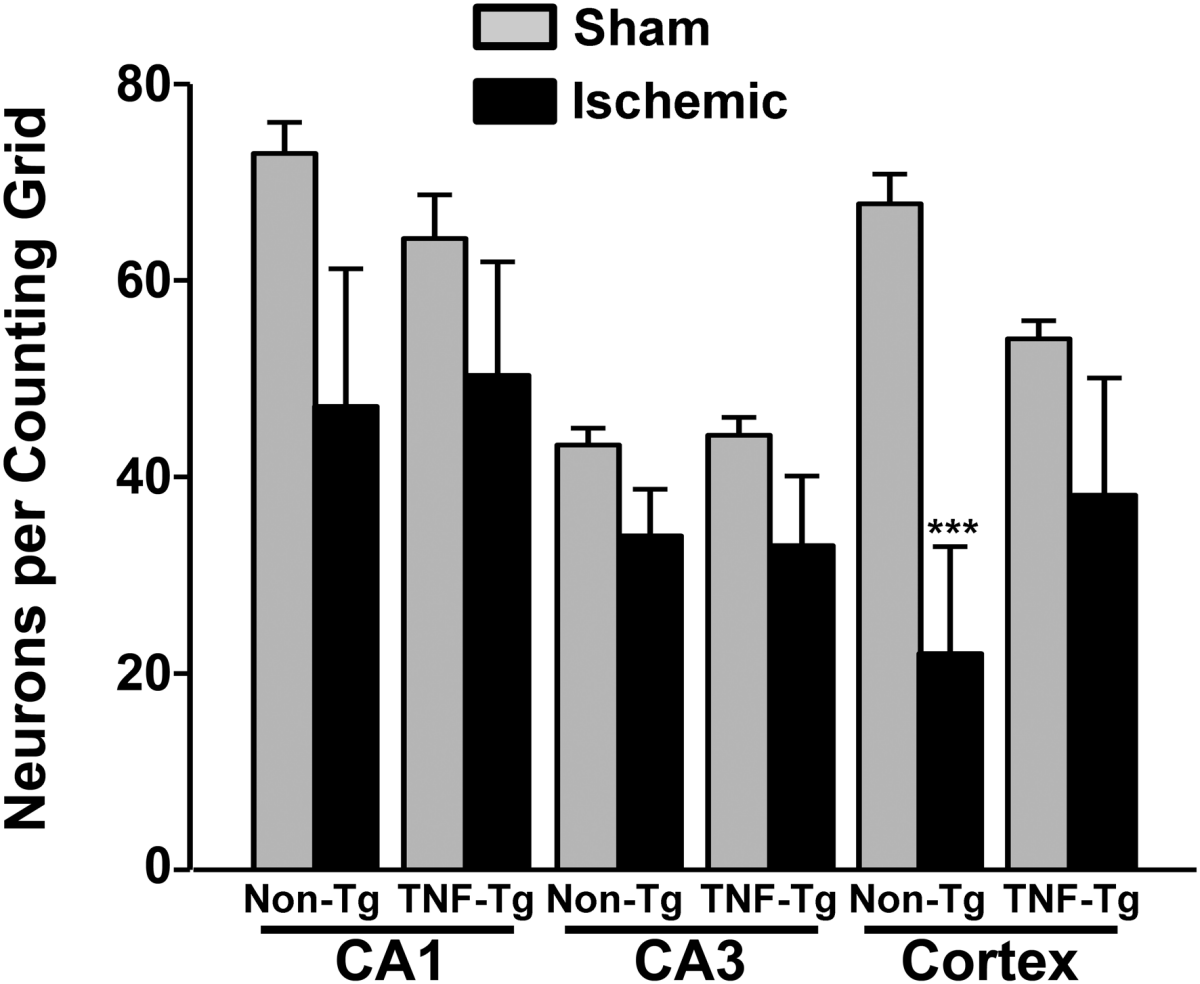
Neuronal loss in hippocampus and cortex of TNFα-transgenic and non-transgenic rats at 28 days after middle cerebral artery occlusion. A bar graph shows Nissl-stained cells per counting grid ± SEM in control (normally perfused) and ischemic hippocampus (CA1 and CA3) and cortex (neocortical layer 5) in TNFα-transgenic (TNFα-Tg) and non-transgenic (non-Tg) littermates subjected to middle cerebral artery occlusion (MCAO). Non-Tg rats had significantly fewer surviving cells in ischemic cortex when compared to the normally perfused hemisphere or when compared to sham operated animals. ***p ≤ 0.001. *n*s = 4 Non-Tg Sham, 8 Non-Tg Ischemia, 3 TNFα-Tg Sham, 6 TNFα-Tg Ischemia.

The results of quantitative histological analysis conducted on TNFα-Tg rats and non-Tg littermates exposed to MCAO or sham surgery are shown in Fig 4. Regional neuron counting was performed on CA1 and CA3 and in cortical layer 5 in both the ipsilateral (*i*.*e*. the side receiving MCAO) and contralateral hemisphere of ischemic TNFα-Tg rats (*n* = 6) and ischemic non-Tg rats (*n* = 8). TNFα-Tg and non-Tg sham-operated rats had similar numbers of pyramidal neurons in CA1, CA3, and cortical layer 5, regardless of hemisphere, and were therefore combined into a single group (*n* = 7) for statistical comparisons to ischemic rats. A one-way ANOVA revealed statistically significant differences in the number of cresyl violet-stained CA1 pyramidal neurons across treatment groups (*p* < 0.05). However, *post hoc* comparisons, found no significant differences between the mean numbers of CA1 neurons sampled from either hemisphere in ischemic TNFα-Tg and non-Tg rats, compared to sham controls. No genotype or ischemia dependent effects were observed for CA3 neuron counts.

The most remarkable differences were observed in pyramidal neuron counts in cortical layer 5, which would be directly affected by ischemic injury. Group variation in layer 5 neuronal count was statistically significant by ANOVA (*p* ≤ 0.001). *Post hoc* comparisons showed significantly fewer cortical layer 5 neurons in the ischemic hemisphere of non-Tg rats (22 ± 11 cells per grid; mean ± SEM) than in the unaffected cortex in the same animals (68 ± 3 cells per grid; Tukey’s test; *p* ≤ 0.001) or in sham-ischemic rats (57 ± 3 cells per grid; p < 0.01). There was no significant difference between the number of surviving cortical layer 5 neurons in the ischemic hemisphere of TNFα-Tg rats (36 ± 12 cells per grid) and those in non-Tg rats. These results suggest that TNF overexpression helps to promote cortical neuron survival following ischemic insult.

## Discussion

We examined indices of synaptic integrity in the TNFα-Tg rat, to determine if this animal has fundamental alterations of hippocampal circuitry that could affect its learning performance. Our data revealed a small nonsignificant decrease in maximal EPSP magnitudes in 8-week TNFα-Tg rats compared to non-Tg littermates. In contrast, measures of short-term and long-term synaptic potentiation (i.e. PPF and LTP) were enhanced in TNFα-Tg rats (Fig 1B–1D). These changes were qualitatively and quantitatively similar to those observed in the hippocampus of immature (< 3-week old) animals, as reported here and previously [52]. In neonatal hippocampus, reduced basal synaptic strength and increased PPF correspond to a reduction in neurotransmitter release capacity [53], while augmented neonatal LTP requires different molecular mechanisms (for review, see [54]) than LTP in mature animals. Intriguingly, adult TNFα-Tg rats also showed impaired performance on hippocampal-dependent behavioral tasks, as discussed below, that are similar to those observed in neonatal animals [55]. Based upon these results, we would hypothesize that elevated brain levels of biologically active TNFα help maintain hippocampal circuitry in a state that mimics early development. Clearly, further studies will be required to fully evaluate this hypothesis.

Though others have previously shown that TNFα can facilitate LTP in hippocampal slices under certain conditions [56], consistent with our findings, several earlier studies reported an impairment in LTP induction in both the dentate and the CA1 regions of the hippocampus following exogenous application of pathophysiological quantities of TNFα [27–29]. These apparent discrepancies may be attributable to several fundamental methodological differences. Most importantly, previous studies investigated acute effects of TNFα on LTP induction (~20 min application). Chronically elevated levels of TNFα in our transgenic animal may recruit transcriptional mechanisms not explored in earlier studies. Also, the synaptic changes observed in the present work may reflect compensatory reactions to the many deleterious effects of TNFα. In addition, earlier studies investigated very long-lasting LTP (~2 hours), whereas we investigated LTP for only one hour after induction. It is possible that deficits in LTP would emerge in the TNFα-Tg rat if synaptic changes were monitored for longer time periods. Finally, it’s important to note that TNF is coupled to divergent signaling pathways via interactions with its different receptor subtypes (TNF-R1and TNF-R2), which could, in turn, result in complex neurologic and cognitive phenotypes. Strategies for teasing apart the differential contributions TNF-Rs to the phenotype of TNFα-Tg rats are considered further below.

We tested spatial reference memory in the TNFα-Tg rat to determine if chronic elevation of TNFα affects cognitive performance before and after focal cerebral ischemia. We found that both TNFα-Tg rats and non-Tg littermates demonstrated successive reductions in AGL during serial training at pre-ischemic baseline, implying progressively more effective learning with repetition in both groups. Transgenic rats showed a trend for more prolonged AGL in comparison to non-Tg littermates, although a statistically significant between-group difference was found only at the fifth and final trial block. While the two groups showed similarly impaired cognitive function at 7 days post MCAO, with overall elevations of mean AGL times compared to pre-ischemic baseline, no significant reduction in AGL over five successive trial blocks, and no between-group difference were detected at any point. However, in probe trial experiments designed to test the accuracy of memory recall, non-Tg rats performed more effectively than TNFα-Tg animals at pre-ischemic baseline and after seven days of post-ischemic recovery.

The incremental, but significant, differences in pre-ischemic performance on the reference memory task suggest that the TNFα-Tg rat showed less effective learning with repetition when compared to non-Tg littermates. Although both animal groups demonstrated similarly impaired learning after being subjected to the physiological stress of ischemia, the probe trial experiments showed that non-Tg rats had more accurate memory recall relative to TNFα-Tg rats. These results indicate that the TNFα-Tg rat showed less effective mastery of reference memory tasks when compared to non-Tg littermates, with or without secondary injury from focal cerebral ischemia. This observation, implying an underlying impairment of cognitive performance in the TNFα-Tg rat, is presaged by an earlier report of impaired learning in two lines of transgenic mice overexpressing TNFα, one of which showed inflammatory changes in the brain and another that showed no inflammatory changes [57]. Overall, the occurrence of retentive memory impairment in our transgenic rat, before and after focal ischemia (and in the transgenic mice with or without active brain inflammation), suggests that chronic overexpression of TNFα alters the molecular basis of learning, even in the absence of cytokine-mediated cellular injury.

We examined performance of the TNFα-Tg rat in the Rotarod task to address two questions: 1) is there an overall difference in functional performance after focal cerebral ischemia that is observed uniquely in the transgenic animal and 2) if present, does this comparative difference persist for an extended duration after ischemia (28 days)? We found that pre-ischemic, mean FL was significantly shorter in TNFα-Tg rats, suggesting that elevated TNF levels precipitate a behavioral phenotype characterized by impaired balance and/or motor coordination. It’s possible that elevated TNF levels adversely affect the vestibular system and/or the neuromusculature, both of which show high vulnerability to multiple autoimmune disorders [58–62]. If so, then it seems likely that this phenotype is overcome or compensated with increased practice, since Non-Tg and TNFα-Tg rats performed similarly on the rotarod during the 28 day testing period following surgery. The effects of elevated TNF on balance and motor coordination would therefore appear to be mild, at least in young adult animals. MCAO had a significant impact on Rotarod performance in both genotype groups, resulting in overall shorter fall latencies. However, ischemic TNFα-Tg rats had shorter, mean test-day FLs for almost twice as many days as non-Tg animals, when compared to sham-ischemic controls. These between-group differences persisted for the 28-day duration of the Rotarod experiment. Again, this suggests that mild deficits in balance and motor coordination are important phenotype traits of the TNFα-Tg rat.

We anticipated that TNFα-Tg rats would have relatively smaller numbers of surviving neurons in ischemic cortex and/or in the CA1 region of ipsilateral hippocampus, when examined 28 days after focal cerebral ischemia as compared to non-Tg littermates. Instead, we found that there were no significant differences in the number of surviving neurons in ischemic cortical layer 5 or in the CA1 region of hippocampus, between the 2 animal groups. The severity of ischemic injury was actually more pronounced in the non-Tg group, in that the number of surviving cortical layer 5 neurons was significantly smaller than in sham-ischemic rats. We speculate that this lack of difference between neuronal counts in ischemic TNFα-Tg rats and non-Tg animals is driven by a “survivor effect” that favored the transgenic animal. In our previous work, we demonstrated that TNFα-Tg rats have significantly greater neural cell apoptosis in ischemic cortex at 24–72 hours relative to non-Tg controls [33]. Before the 28-day, post-ischemic interval, evaluated in the present study, the synergistic interaction between chronically elevated regional TNFα and ischemic physiological stress may have already exerted its maximal effect on cellular apoptosis in ischemic cortex and hippocampus. Consistent with this idea is abundant evidence showing the importance of TNFα in mediating inflammation and apoptosis, characteristic of most neurodegenerative diseases. In vascular cognitive impairment (VCI) and other disorders resulting from cerebral ischemia, TNFα (expressed by vascular and perivascular cells) can promote pro-inflammatory and pro-coagulant effects on the endothelia [63]. In a rat model of VCI, produced by surgical occlusion of both carotid arteries, subcortical vessels become immuno-reactive for TNFα [64, 65]. Nasal administration of recombinant E-selectin, a glycoprotein adhesion molecule that stimulates the synthesis of regulatory T-cells targeting activated endothelia, suppresses TNFα vascular immuno-reactivity in this model. Moreover, induction of mucosal tolerance to E-selectin prevents ischemic and hemorrhagic stroke in spontaneously hypertensive stroke-prone rats [66] and reduces infarct volume after permanent MCAO [67], suggesting that the deleterious effects of vascular pathology are mediated, at least partly, through the activation of TNF signaling mechanisms.

As alluded to above, TNF can mediate complex phenotypic effects through actions on its different receptor subtypes. Soluble TNFα interacts preferentially with type TNF-R1s, while transmembrane TNFα shows greater selectivity for TNF-R2s [68, 69]. In turn, TNF-R subtypes are coupled to divergent downstream signaling pathways: TNF-R1 activation stimulates caspase activity and cell death, while TNF-R2 activation stimulates PI3-kinase signaling and promotes cell survival [70]. It is widely believed that many of the detrimental pro-inflammatory actions of TNF are mediated by TNF-R1s, while several of the more beneficial actions of TNF are carried out by TNF-R2s [70]. Presently, it is unclear to what extent TNF-R1 and TNF-R2 signaling is engaged in TNFα overexpressing rats, or how these different receptor mechanisms impact the cellular and behavioral phenotypes investigated in this study. Biologics that preferentially target different TNF-Rs have been used in other model systems to tease apart the relative contributions of soluble and transmembrane TNF. For instance, XPro1595 is a soluble TNF mimetic that exhibits dominant-negative inhibition of TNF-R1, but not TNF-R2, signaling [71]. This drug has revealed selective deleterious roles of TNF-R1 in animal models of Parkinson’s disease [72, 73], Alzheimer’s disease [74], and autoimmune encephalomyelitis [75], Previously, we used XPro1595 and a variety of biochemical approaches to show that soluble TNF/TNF-R1 interactions contributes to cognitive decline, neuronal Ca^2+^ dysregulation, and altered synaptic plasticity in aged rats [34]. Future use of XPro1595, or similar reagents, could therefore be a powerful way to dissect apart the apparently complex phenotype of TNFα overexpressing rats.

Finally, changes in glial activation and inflammatory profiles were not investigated in the present study, and as such, it is unclear whether the phenotypic traits reported in TNFα overexpressing rats are attributable to direct effects of TNF on neuronal signaling pathways, or to indirect effects on glial cells (both microglia and astrocytes), which can alter neuronal function through a myriad of mechanisms. Indeed, TNF can cause profound glial activation, which has been noted in similar TNFα overexpressing mouse models [76, 77]. Numerous glial-derived cytokines that are induced or potentiated by exogenously applied TNF can significantly modulate synaptic function, neuronal morphology, and neuronal survival/death pathways [78–82]. In addition, TNF application to both astrocytes and microglia can cause the release of numerous reactive oxygen species known to erode synaptic efficacy and/or to precipitate neurodegeneration [83, 84]. Finally, TNF and related cytokines have been shown to negatively affect numerous protective properties of astrocytes, including glutamate uptake [85, 86] and gap junction coupling [87], to name a few. All of these mechanisms could be in play in shaping the neurologic and cognitive phenotype of TNFα overexpressing rats and should be investigated in future studies.

## Conclusions

Our results suggest that elevated brain levels of TNFα, similar to that found in a number of neurodegenerative disorders, can contribute to impaired cognition and functional performance. Our LTP studies in non-ischemic TNFα-Tg rats suggest that constitutive upregulation of TNFα synthesis may facilitate maintenance of hippocampal circuitry in a nascent stage. Although such a fundamental alteration could be beneficial in preserving normal hippocampal function under the physiological stress of cerebral ischemia, we found that post-ischemic TNFα-Tg rats showed impaired performance on both cognitive and functional tasks at extended time intervals after MCAO. By histological analysis, this impairment was not associated with excessive neuronal loss from the CA1 or CA3 regions of hippocampus or from post-ischemic cortex. We cannot exclude the possibility that constitutive upregulation of TNFα synthesis produces other alterations in the transgenic rat that could influence performance on cognitive and functional tasks, such as the release of inhibitory or excitatory neurotransmitters or the modulation of cortico-hippocampal connections. Overall, our results substantiate the impression that increased brain synthesis of TNFα will precipitate long-term cognitive effects and contribute to higher risk of dementia after cerebral ischemia.

## Supporting Information

S1 File Raw data for producing figures and for conducting statistical analyses. (XLS)
